# A Case of Laparoscopic Excision of a Huge Retroperitoneal Cystic Lymphangioma

**DOI:** 10.1155/2011/712520

**Published:** 2011-07-14

**Authors:** Yusuke Yagihashi, Keiji Kato, Kanji Nagahama, Masakazu Yamamoto, Hiroshi Kanamaru

**Affiliations:** Department of Urology, The Tazuke Kofukai Medical Research Institute, Kitano Hospital, Osaka 530-8480, Japan

## Abstract

Retroperitoneal cystic lymphangioma is a rare benign tumor. Most patients eventually experience some symptoms that necessitate therapeutic intervention. Excision is the treatment of choice, and some cases of laparoscopic resection have been reported. We report another case of a huge retroperitoneal cystic lymphangioma that was successfully excised laparoscopically with the SAND balloon catheter. Large cystic lymphangioma was downsized by puncturing and aspirated with the SAND balloon catheter. Laparoscopic surgical technique should be considered for treatment of selected cystic lesions of retroperitoneal origin.

## 1. Introduction

With the recent development of surgical equipment and advance of surgical techniques, numbers of minimally invasive procedures with laparoscopy for treatment of large tumors are advocated. This paper describes a patient with a huge retroperitoneal cystic lymphangioma who underwent a successful laparoscopic resection. 

## 2. Case Report 

A 38-year-old woman without any relevant medical history was admitted to our hospital with an abdominal tumor that had caused recurrent left hypochondrial pain for 6 months. 

A physical examination revealed a tense mass located in the lower abdomen. Ultrasonography revealed left hydronephrosis and a huge retroperitoneal cyst. 

**Figure 1:**
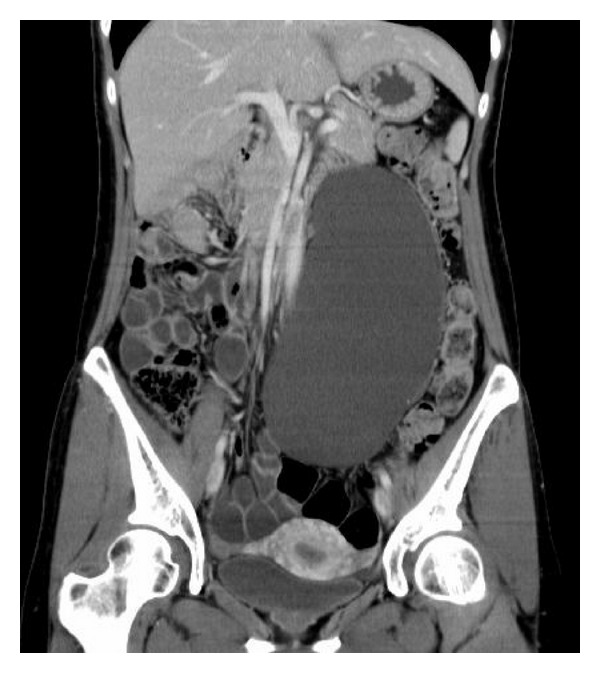
Coronal section of abdominal CT demonstrating a large water density occupying much of the left retroperitoneal space.

Abdominal CT scan revealed a retroperitoneal mass and spreading from level of common iliac vessels up to renovascular level and left hydronephrosis (Figure 1).

**Figure 2:**
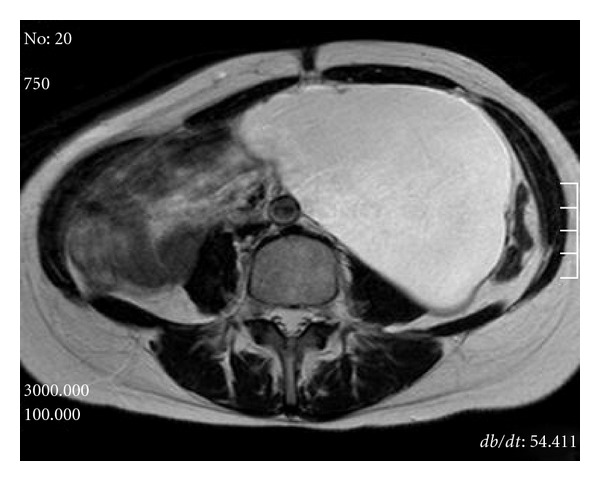
Magnetic resonance imaging showing a large simple cystic retroperitoneal mass.

**Figure 3:**
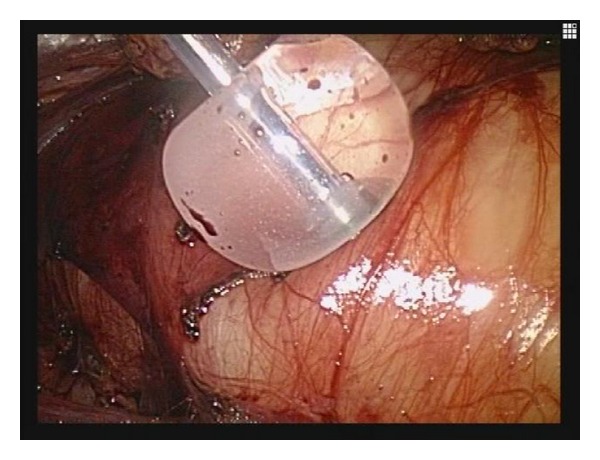
Laparoscopic view showed a wall of the retroperitoneal lymphangioma. Only the inflated proximal balloon is visible.

MRI showed a monolocular cyst of measuring 18 cm in diameter without solid component (Figure 2). The wall of the mass was thin and smooth. Radiological diagnosis of retroperitoneal lymphangioma was established. Tumor markers including carcinoembryonic antigen, CA19-9, CA125, and alpha-fetoprotein were measured to assist the diagnosis. Routine laboratory and hematologic investigations were unremarkable and laparoscopic excision was performed because of the patients′strong desire for cosmetic surgery. We decided to do a diagnostic and therapeutic laparoscopy. Preoperative informed consent including a statement that laparotomy might be required if the mass could not be managed by laparoscopy was obtained from the patient and family. The patient was placed in the lateral decubitus position. Four trocar placement in the retroperitoneal approach, the primary site for the telescope is placed between the tip of the 12th rib and the iliac crest, and pneumoretroperitoneum is created and three additional ports were inserted. The second port is placed 7 cm medial and slightly superior to the primary site in the anterior axillary line. The third port was placed close to the costovertebral angle. The fourth port may be used for retraction and is placed in the anterior axillary line, about 7 cm inferior to the working port. The dissection was accomplished easily. However, after half of cyst wall was dissected from retroperitoneum, it became difficult to dissect. Therefore the SAND balloon catheter (Hakko Medical, Tokyo, Japan) through the fourth port was stabbed into the tumor and utilized to aspirate the fluid content (Figure 3). The punctured cyst wall is sandwiched between the two inflated balloons. The tumor was aspirated carefully using a SAND balloon catheter to reduce the size without spilling of the tumor content under laparoscope. About 1 litre of fluid was removed from the cyst. During the maneuvers of mobilization and as a consequence of the fragile wall, the cystic tumor was opened and the fluid was suctioned. The tumor pedicle is coagulated and cut by LigaSure V (Tyco Healthcare Japan, Tokyo, Japan). The excised tumor was put into an Endopouch retriever and removed from the body. The cyst could be completely removed. And its wall was thin without any solid elements. Analysis of the fluid revealed lymph. Cytology showed no malignant cells. Surgical duration was 290 minutes and the estimated blood loss was less than 10 mL. Histological examination confirmed the preoperative clinical and radiological diagnosis. A physical examination revealed complete resolution of the abdominal swelling and the hydronephrosis resolved after the operation. No recurrence has been noted on radiographic imaging 16 months postoperatively.

## 3. Discussion

Cystic lymphangiomas are congenital benign tumors of the lymphatics. The histogenesis of lymphangioma is still uncertain. More than 95% of cystic lymphangiomas occur in the head, neck, and axilla with only 1% in the retroperitoneum. Lymphangiomas have been classified as simple, cavernous, and cystic according to their histologic appearance [1–3]. The most characteristic radiologic finding of the retroperitoneal lymphangioma is a large tumor containing uncomplicated fluid with or without septa. At CT, cystic lymphangioma appears large and thin walled. MRI usually demonstrates the typical signal changes of a fluid-filled cyst. The differential diagnosis of a retroperitoneal cyst includes a teratoma, ovarian cystadenomas, retroperitoneal hematomas, abscess, malignant fibrous histiocytoma, and liposarcoma. However, establishing a correct preoperative diagnosis with imaging diagnostic modalities is frequently difficult. Surgical excision is the treatment of choice because of its potential to grow and develop complications. When they become large enough, they can compress the bowel or urinary tract and present as abdominal or back pain. If surgical excision is used in treatment, it needs to be as complete as possible in order to reduce the risk of recurrence. When treating a huge retroperitoneal lymphangioma, laparotomy was chosen in previously cases [2–4]. However, this open procedure often results in greater postoperative pain and poor cosmetic appearance. When management of large cystic retroperitoneal tumors with minimal incision is intended, downsizing the tumor volume by puncture of cyst and aspiration of cystic contents with minimum spillage is required to permit mobilization and removal of the tumor. Spillage of the cyst contents into the retroperitoneal cavity may occur during aspiration when an ordinary aspiration needle is used. We therefore use a specially designed double-balloon catheter for aspiration to minimize spillage of the cyst contents into the retroperitoneal cavity. This balloon catheter is developed mainly for laparoscopic cystectomy of benign ovarian cysts [5].

**Figure 4:**
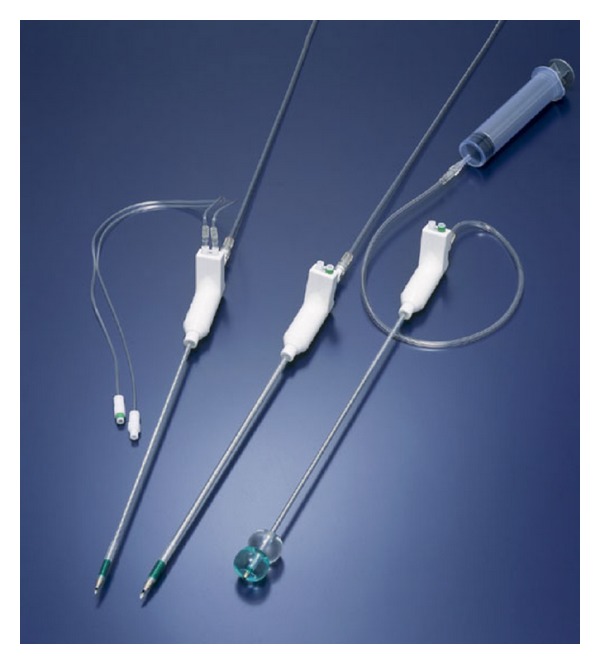
Structure of the SAND balloon catheter.

This catheter consists of internal and external catheters. The internal catheter has a needle for a cyst puncture at its tip. The needle can be removed after the cyst is punctured. The external catheter has two balloons near its tip. Each balloon can be separately inflated with 10 mL of physiological saline (Figure 4). Spillage of the cyst contents can be minimized by sandwiching the punctured cyst wall between the inflated distal and proximal balloons. Furthermore, this catheter also functions as a grasper and retractor. However, because minimal spillage is not completely prevented, even with careful use of the SAND balloon catheter, laparoscopic monitoring is important to assess the degree of accidental spillage of tumor contents. Although not a few cases treated laparoscopically were reported [6–10], our report is still worth in terms of using a specially designed double-balloon catheter to minimize the spillage of cystic content and reduce the size under laparoscope.

Laparoscopic surgery with the SAND balloon catheter for a huge retroperitoneal lymphangioma is a safe and minimally invasive procedure and cosmetically effective.

## References

[1] Meyer T, Stöhr G, Post S, Fayyazi A (1995). Retroperitoneal lymphangioma presenting as a mesenteric cyst. *European Journal of Radiology*.

[2] Hayami S, Adachi Y, Ishigooka M (1996). Retroperitoneal cystic lymphangioma diagnosed by computerized tomography, magnetic resonance imaging and thin needle aspiration. *International Urology and Nephrology*.

[3] de Perrot M, Rostan O, Morel P, Le Coultre C (1998). Abdominal lymphangioma in adults and children. *British Journal of Surgery*.

[4] Richmond B, Kister N (2009). Adult presentation of giant retroperitoneal cystic lymphangioma: case report. *International Journal of Surgery*.

[5] Yamada T, Okamoto Y, Kasamatsu H (2000). Use of the SAND balloon catheter for the laparoscopic surgery of benign ovarian cysts. *Gynaecological Endoscopy*.

[6] Targarona EM, Moral A, Sabater L, Martínez J, Luque P, Trías M (1994). Laparoscopic resection of a retroperitoneal cystic lymphangioma. *Surgical Endoscopy*.

[7] Tsukamoto T, Tanaka S, Yamamoto T (2003). Laparoscopic excision of a retroperitoneal cystic lymphangioma: report of a case. *Surgery Today*.

[8] Wildhaber BE, Chardot C, Le Coultre C, Genin B (2006). Total laparoscopic excision of retroperitoneal cystic lymphangioma. *Journal of Laparoendoscopic and Advanced Surgical Techniques*.

[9] Singh RR, Govindarajan KK, Bowen C, Chandran H (2009). Retroperitoneal cystic lymphangioma: a rare presentation in childhood, treated laparoscopically. *Journal of Laparoendoscopic and Advanced Surgical Techniques*.

[10] Kasza J, Brody FJ, Khambaty F, Vaziri K, Wallace B (2010). Laparoscopic resection of a retroperitoneal cystic lymphangioma in an adult. *Surgical Laparoscopy, Endoscopy and Percutaneous Techniques*.

